# Mr. Earnest's Singular Case of Costiveness

**Published:** 1806-02

**Authors:** Robert Earnest

**Affiliations:** House Surgeon to the Sheffield General Infirmary


### *To the Editors of the Medical and Physical Journal.*

GENTLEMEN,

THE enclosed is a singular case of costiveness, wherein the more powerful cathartics had little or no effect. The complaint became habitual, by the inattention and indolence of the patient postponing and retarding, for four or five days, and sometimes for a week together, the natural efforts of the rectum, &c. to evacuate the fæces. If, after a perusal, you should deem the case worthy a place in your useful Journal, you will greatly oblige me, by inserting it in the next number.

I am, &c.

*Jan.* 10, 1806.

ROBERT EARNEST,

House Surgeon to the Sheffield General Infirmary.

---

THE subject of this case is a girl seventeen years of age, of a strong, robust, athletic constitution, who had been troubled with costiveness (brought on in the way above-mentioned) for six or seven months. She had very seldom more than one evacuation in eight or ten days. She had menstruated regularly. The symptoms attending this constipated state of the bowels, were as follows, viz. head-ach, flatulence in the stomach and bowels, accompanied with pain and nausea; the abdomen was much tumefied; the lower extremities were in a general way cold; loss of appetite; pulse 70. She only passed off small quantities of urine, but she perspired freely. She had been affected in this way nearly seven months, when the symptoms were aggravated by a sudden check to the catamenia, which was caused by being exposed to the wet and cold, for nearly a whole day. It was now that I first saw the patient, Oct. 16, 1805. I bled her from the arm, and ordered leeches to the feet, and afterwards a pediluvium. She was now put upon a variety of powerful purgative medicines, which were persisted in for five days, namely, from the 16th to the 21st of October, but without effect.

To enumerate all the different formulæ, which were prescribed during these five days, in their successive order, would be trespassing too far; and would also extend the case to an unnecessary length. I think it right notwithstanding, to mention, without putting them into their proper prescriptive form, the quantities of the different purgative gative medicines, which the patient took in the space of time just specified, namely, Magnesia vitriolata unc. iv. Tinct. senna, unc j. Tinct. aloes, unc j. Tinct. jalap, dr. vj. Pulv. jalap, ℈ij. Extract. colocy. ℈ij. Calomel, ℈j. Aloes and gamboge of each four grains as a bolus. Cremor. tartar. unc ij. Ol. ricini, unc viij. Infusum sennæ tartarisatum, unc viij.*

During the exhibition of all these medicines, purgative glysters also, and the general warm bath were used; and the result arising from all these remedies, will appear almost incredible, when I say, that only two evacuations were produced, which were still of a costive nature. The nausea was increased, and consequently the appetite was more impaired by them; the urine was also increased in quantity, but the perspiration was diminished. Although the nausea was so constantly troublesome, yet the stomach never rejected any one of the medicines that were given.

Oct. 22. All drastic cathartics were now ordered to be omitted, and in place of which the patient took a table-spoonful of castor oil every two hours; and which she continued regularly to take until October 31. This medicine proved no more effectual than any of the other before-mentioned, as the action of the bowels were still immoveable.

After taking four or five ounces of the ol. ricini, daily, for nine days together, the patient passed only one stool, and that happened on the ninth day from the beginning.

Nov. 1. As purging medicines were of no avail, they were left off altogether.

Nov. 3. The patient passed two natural stools, and from this time the bowels became regular, and are so at this present time. The nausea at the stomach gradually went off, and the appetite as gradually increased, and the girl is now in perfect good health.

### REMARKS.

It appears in this case of obstinate costiveness, that powerful cathartics had little or no effect, and to account for their inefficacy is perhaps no very easy task, and requires a much greater share of physiological knowledge

 than

* As it will appear very strange that these medicines were so unsuccessful in their usual mode of operating, some blame may be attached to them, doubting the goodness of their quality; but I affirm, that they were certainly genuine.

than I pretend to possess, to enable me to explain the principal cause of this long continued and obstinate complaint. However, I will here venture to offer a few conjectural remarks upon the case.

1. The catamenia having been checked by such powerful sedatives as wet and cold, I conceive had the effect at such a critical time, of bringing on spasms,* not only in the uterine vessels, but also in the stomach and bowels, &c. and that the same cause had a share in further constricting the bowels, and thereby aggravated the predisposition to such a complaint; whence also might arise pain, flatulence, fullness of the abdomen, coldness of the legs and feet, &c.

2. That the same efficient cause very probably diminished in some degree the irritability of the stomach and bowels, and other parts at the same time affected, which would in all likelihood tend in some measure also to act in opposition to the usual effects of such remedies.†

3. Costiveness prevailing for such a length of time, shews also a great defect of the peristaltic motion of the bowels.‡

4. Although cathartics in this case appeared to be the most rational indication of cure, yet they proved detrimental to the operations of nature; for they had not been left off more than three days, before the menstrual discharge appeared again; and now, as it were, the bowels spontaneously became soluble and regular.

5. It is well known that persons with strong and robust constitutions, and who are said to be of the rigid fibre, are much more slowly acted upon by medicine than those with weakly constitutions, who are said to be of the lax fibre. This patient being of the former stamina, may also be accountable in some respects for the non-effect of the medicines.

6. This patient at the origin of her complaints, may have been one of that class of people spoken of by Baron Van

---

* Hoffman says, "That costiveness is generally owing to spasms in the intestines themselves, or as propagated by consent; but various causes conduce to this habit, as an inert bile, acidity prevailing greatly in the first passages, coldness of the feet, &c."

† "It is said that the effects of medicines are very often to be deduced from this irritability, as they depend on either increasing or diminishing it."

‡ "It is also said, that the peristaltic motion of the intestines is not constant, but takes place on proper occasions, or as these bowels are stimulated by their contents, &c."

*Motherby's Medical Dictionary, see Irritabilitas & Intestina.*

Van Swieten, in his Commentaries upon Boerhaave's Aphorisms, vol. v. p. 257.—"Some lean people of a tense fibre, have such strong chylificative viscera, that they draw off every thing that is soluble from the ingested aliments, and therefore leave the fæces accumulated, dry, and juiceless, in the large intestines; and as in such dry habits there is found a less quantity of that lubricating mucus lining the large intestines, especially towards the extremity of them, therefore the exclusion of dry fæces is rendered more difficult; hence such people are commonly costive, and frequently to a very great degree."

7. Perhaps, had antispasmodics and cathartics been conjoined, along with the use of the warm bath, their general remedial powers, might have proved more effectual and beneficial.

